# Multi-compartment encapsulation of communicating droplets and droplet networks in hydrogel as a model for artificial cells

**DOI:** 10.1038/srep45167

**Published:** 2017-04-03

**Authors:** Mariam Bayoumi, Hagan Bayley, Giovanni Maglia, K. Tanuj Sapra

**Affiliations:** 1 Department of Chemistry, KU Leuven, Celestijnenlaan 200G, 3001 Leuven, Belgium; 2 Chemistry Research Laboratory, 12 Mansfield Road, Oxford, OX1 3TA, United Kingdom; 3 Groningen Biomolecular Sciences and Biotechnology Institute, University of Groningen, Nijenborgh 7, 9747 AG Groningen, The Netherlands; 4 Department of Biochemistry, University of Zurich, Winterthurerstrasse 190, 8057 Zurich, Switzerland

## Abstract

Constructing a cell mimic is a major challenge posed by synthetic biologists. Efforts to this end have been primarily focused on lipid- and polymer-encapsulated containers, liposomes and polymersomes, respectively. Here, we introduce a multi-compartment, nested system comprising aqueous droplets stabilized in an oil/lipid mixture, all encapsulated in hydrogel. Functional capabilities (electrical and chemical communication) were imparted by protein nanopores spanning the lipid bilayer formed at the interface of the encapsulated aqueous droplets and the encasing hydrogel. Crucially, the compartmentalization enabled the formation of two adjoining lipid bilayers in a controlled manner, a requirement for the realization of a functional protocell or prototissue.

Synthetic biology seeks to build cells and modify them to understand how life began, functions, and evolves^1^,^2^, as well as to engineer and exploit new life forms^3^,^4^. The top-down approach boasts of synthesizing a minimal genetic blueprint^5^,^6^,^7^, ‘creating’ a cell^8^, and taming metabolic pathways for biotechnological applications^9^,^10^,^11^. With equally grand ambitions, bottom-up synthetic biology is focused on the *de novo* design of a cell^12^,^13^ with the specific aims of building minimal structures^14^,^15^ and mimicking complex cellular functions^16^,^17^,^18^,^19^. Toward the realization of synthetic cellular systems, success has been achieved in the bottom-up design of a protocell^20^, and intriguing possibilities have been demonstrated for a functional prototissue^21^,^22^.

The success of the biological cell depends on compartmentalization. A direct consequence of compartmentalization is chemical and electrical signaling, which are key factors in imparting emergent properties to biological cells and tissues. Consequently, a mandatory feature of a protocell, and the success of its translation into a prototissue, is compartmentalization and communication between its multiple compartments^23^. As in natural cells, enclosing DNA, RNA, and proteins within protocells ensures protection from degradation^24^ while providing the required concentrations for optimal function^25^,^26^. For protocellular systems, delimiting the active contents from their environment bestows the possibility of functional engineering^27^ by means of spatial^28^ and temporal control over the system^22^,^29^.

In recent years, protocells have been introduced for applications in drug delivery and nanotechnology^30^ (e.g., nanometer-sized lipid vesicles^31^, giant unilamellar vesicles^32^, polymersomes^33^, capsosomes^34^, proteinosomes^35^, vesosomes^36^). Recently, aqueous droplets in oil have been proposed as protocell models^37^. The droplet protocells in an oil/lipid bath are connected through lipid bilayers at the contact interfaces^38^. Bilayer-linked aqueous droplets in a network are capable of electrical and chemical communication with each other^39^ and with surrounding aqueous medium^40^ *via* protein nanopores. Droplet networks have been shown to exhibit emergent properties of electrical^41^ and mechanical nature^21^, the first steps toward formation of a prototissue. The aqueous droplets can be replaced by millimeter-sized hydrogel pieces in oil with stable bilayers at their interfaces^42^,^43^.

Here, we incorporated aqueous droplets, stabilized in an oil/lipid bath, inside a hydrogel, which might serve as the basic unit for the bottom-up construction of a protocell, and a collection of these might make prototissue (Fig. 1). The use of a firm hydrogel matrix was key to forming multiple compartments inside the same hydrogel unit. The stable encapsulation of aqueous droplets in different oil compartments held in the hydrogel enabled the formation of two bilayers close to each other – a first step toward engineering organelles and cell mimics for controlled electrical and chemical communication. A major advantage of the present strategy is the ease of hierarchical encapsulation, thereby offering a clear demarcation between a proto-organelle, a protocell and a prototissue.

## The concept

**Figure 1:**
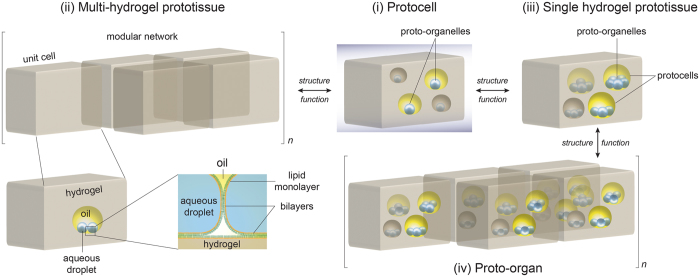
Concept of bottom-up design of artificial cells and modular tissues. Akin to a biological tissue, we define an ensemble of protocells as a prototissue, and a network of prototissues is considered a proto-organ. A simple bottom-up hierarchical construction would be – proto-organelle → protocell → prototissue → proto-organ. The proto-organelle is simply an aqueous droplet immersed in an oil/lipid bath to coat it with a lipid monolayer. To produce a system with hierarchical properties, the proto-organelle in oil is encased in a hydrogel. The aqueous droplet-hydrogel interface is stabilized by a lipid bilayer capable of functionalization with membrane proteins (e.g., chemical and electrical communication, sensing). Because protocells contain proto-organelles, *multi-compartment* encapsulation of *single* aqueous droplets (proto-organelles) in a hydrogel will form a protocell (i). An assembly of such hydrogel-protocells, ideally connected through lipid bilayers^42^, will form a prototissue (ii). Alternatively, *multiple* aqueous droplets (proto-organelles) in the same oil compartment (encased in hydrogel) will also constitute a protocell; multi-compartmentalization of the protocells in a hydrogel will constitute a prototissue (iii). A collection of such prototissues would be a proto-organ (iv). The crucial element of the proposed system is multiple levels of compartmentalization, modularity and spatial flexibility. The modular design has the advantage of structure-function interconversion; an agarose unit can be a proto-organelle, a protocell or a prototissue. Because the hydrogel pieces can be spatially manipulated^42^, a protocell can be introduced into or removed from a prototissue, e.g., by adding or removing a piece of hydrogel (i ↔ ii); injecting aqueous droplets can convert a protocell into a prototissue (i → iii); and prototissues can be assembled into or removed from a proto-organ (again by adding or removing a piece of hydrogel) (iii ↔ iv).

We started with the premise that the scaffold of a protocell should also act as the basic unit of a prototissue in a true hierarchical sense [proto-organelle → protocell → prototissue (→proto-organ)]. For both a protocell and a prototissue, a crucial feature would be the presence of a viscous fluid or a gel-like material between the proto-organelles (similar to cytoplasm), and between the protocells (mimicking the extracellular matrix), respectively. A hydrogel framework, encapsulating aqueous droplets in a single or multiple oil drops, served the purpose. Depending on the number of oil and aqueous droplets included, the module can either be a protocell or a prototissue (Fig. 1). For example, a single aqueous droplet in oil would be a proto-organelle; many proto-organelles housed in the same hydrogel but in different oil enclosures would constitute a protocell; (i) in Fig. 1. Many such protocells when connected through bilayers would form a multi-hydrogel prototissue; (ii) in Fig. 1^42^. Another way of constructing a prototissue is to incorporate a number of protocells in the same hydrogel. A protocell in this scenario is made by a collection of aqueous droplets in an oil drop, each of the aqueous droplets being proto-organelles; (iii) in Fig. 1. A key difference between the proto-organelles formed in (ii) and (iii) is the nature of the lipid bilayer. Whereas in (ii) a proto-organelle has an individual communication line (bilayer), in (iii) the proto-organelles can perform more complex communication patterns, because the same bilayer is shared by at least two proto-organelles. A network of prototissues as in (iii) could be the basis of mimicking an organ; (iv) in Fig. 1.

The focus of this work is to demonstrate the stable encapsulation of one aqueous droplet (proto-organelle) or many aqueous droplets (protocell) in hydrogel, the module serving as the basic structural and functional unit in the bottom-up construction of a prototissue.

## Results

### Encasing aqueous droplets in hydrogel

**Figure 2:**
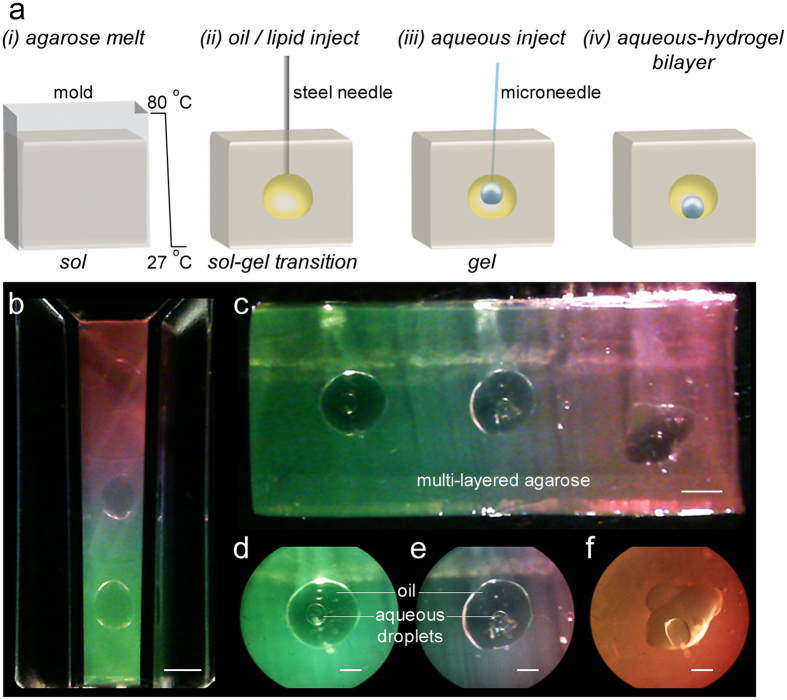
Multiple encapsulation of aqueous droplets (proto-organelles) in a hydrogel to form a protocell. (**a**) Low gel agarose (1% w/v in 150 mM NaCl, 10 mM Tris-HCl, pH 7.5) was melted by heating to 80 °C, poured into a mold, and allowed to cool down to 27 ± 1 °C (*i*). At this sol-gel transition point 10–100 μL of hexadecane/silicone oil mixture containing 7.5 mg mL^−1^ DPhPC was injected inside the hydrogel using a steel needle, which was retracted without delay (*ii*). The oil drop was firmly encased as the agarose completely gelled upon reaching the room temperature (~22 °C). Aqueous droplets were then injected into the oil using a microneedle (*iii*). The aqueous droplet settled at the bottom of the oil drop stabilized by a lipid bilayer (indicated by the stability of the aqueous droplets and an increase in the bilayer capacitance, Supplementary Figures 2 and 4, respectively) at the aqueous-hydrogel interface (*iv*). (**b**) A 3-tier agarose block was formed in a cuvette (colored with water soluble dyes; bottom layer with pyranine, the middle layer without any dye, and the uppermost layer with rhodamine). The hydrogel layers were formed sequentially encapsulating an oil drop after each layer was formed. (**c**) The hydrogel block could be easily removed from the cuvette after complete gelation, and placed in a Petri dish with oil covering the hydrogel. The oil drops were then accessible for injecting aqueous droplets in different compartments using a microneedle giving a protocell (cf. (i) in Fig. 1). (**d**–**f**) Zoom in of aqueous droplets in the 3 oil chambers. Scale bars, 1 mm.

**Figure 3:**
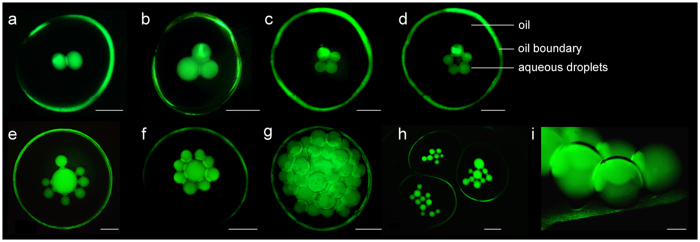
Assemblies of aqueous droplets in hydrogel as protocell models. Stable aqueous droplet assemblies (appear as solid green spheres because of pyranine) made of, (**a**) 2, (**b**) 3, (**c**) 4, (**d**) 5 droplets in hydrogel-encased oil drops (the green circles are owing to the reflection of pyranine and demarcate the oil boundaries). Each of these oil drop enclosures can be viewed as a protocell where the aqueous droplets are the proto-organelles. (cf. (iii) in Fig. 1). (**e**,**f**) The droplet sizes and the numbers could be varied. (**g**) A 3D assembly of aqueous droplets containing >40 droplets (Supplementary Figure 1). (**h**) Three oil drops in a single piece of hydrogel containing aqueous droplet networks of different geometries. This collection of protocells can be regarded as a prototissue model (cf. (iii) in Fig. 1). (i) Side-view showing aqueous droplets interfacing with an agarose surface through stable lipid bilayers at the aqueous-hydrogel interface. The aqueous droplets contained 10 mM pyranine (green colour). Scale bars, (**a**–**h**) 1 mm; (**i**) 100 μm.

Owing to its ease of availability, low cost, and biocompatibility, agarose was chosen as the hydrogel matrix for droplet encapsulation. Crucially, the oil drop (a 1:1 v/v mixture of hexadecane and silicone oil with the lipid DPhPC) was injected precisely at the sol-gel transition temperature (27 °C) of the agarose (Fig. 2a, see Methods). Nanoliter volumes of aqueous droplets containing liposomes were injected into each of the oil compartments using a microneedle (Supplementary Movie 1). The droplets settled at the bottom of the oil chamber forming a lipid bilayer at the aqueous-oil-hydrogel interface^44^. Such an aqueous droplet in oil is a proto-organelle, and a collection of such proto-organelles in the hydrogel a protocell. Figure 2 demonstrates the construction of a protocell comprising three proto-organelles. Fig. 3 shows examples of other protocells with 2D networks of aqueous droplets, and a stable 3D network of aqueous droplets (Supplementary Figure 1)^21^ inside an oil compartment encapsulated in hydrogel. Aqueous droplet networks in three separate oil compartments, i.e., protocells, encapsulated in the same hydrogel form a prototissue (Fig. 3h, cf (iii) in Fig. 1).

### Encapsulated aqueous droplets form stable bilayers

The hydrogel-encapsulated aqueous droplets were stable, i.e., they did not fuse with the hydrogel when subjected to mechanical motion. For example, carrying the hydrogel piece from one lab to another (~25 m), or rotating the hydrogel did not destroy the aqueous droplets. Aqueous droplets in different assemblies (up to 8 droplets) inside the oil chamber were stable for up to 80 h at 29 °C, while the stability was marginally lower at 37 °C (Supplementary Figure 2). The droplet stability may be attributed to lipid bilayer formation at the aqueous-hydrogel interface^44^. The lipids in the oil coat the inner surface of the hydrogel enclosure to form a lipid monolayer; the aqueous droplets containing liposomes are too encased by lipid monolayers when injected into the oil^37^. Upon settling on the lipid monolayer-coated hydrogel surface, the contact interface between the aqueous droplets and the hydrogel is stabilized by the formation of a lipid bilayer (Figs 1 and 2). This stabilization may be attributed to reaching a free energy minimum in a complex energy landscape as in the case of multisomes^40^.

**Figure 4:**
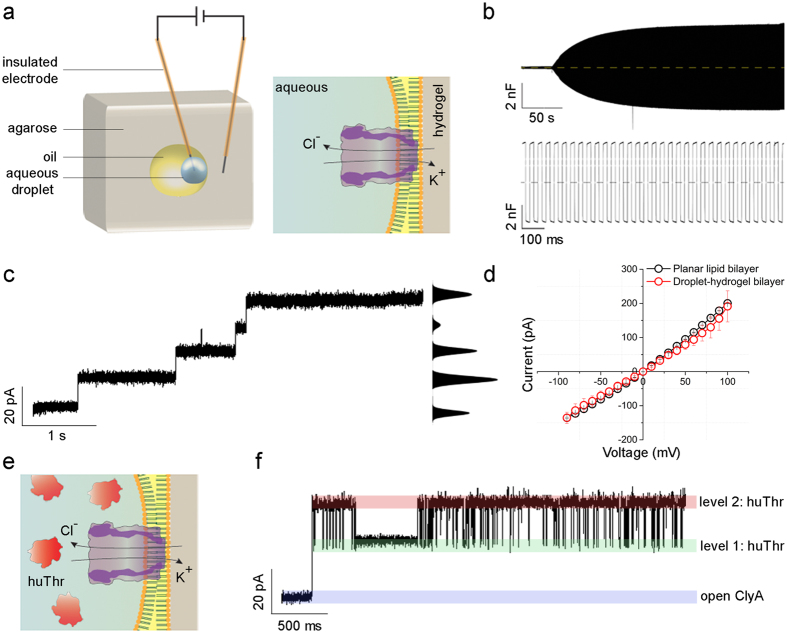
Encapsulated droplets in hydrogel (proto-organelles) are functional. (**a**) Lipid-monolayer encased aqueous droplets encapsulated in hydrogel were capable of forming stable bilayers at the aqueous-hydrogel interface as determined electrically by an increase in the electrical capacitance (**b**). (**c**) The bilayers were conducive to the insertion of membrane pores like α-hemolysin (not shown) and ClyA observed as a step-wise increase in the electrical current. (**d**) The I-V curve of ClyA in an encapsulated proto-organelle bilayer was similar to that in a planar lipid bilayer confirming the stable insertion of the pores in the hydrogel platform. (**e**,**f**) The proper assembly of ClyA was further ascertained by including human thrombin (huThr) in the aqueous droplet with ClyA. The transient kinetics of huThr blocking were similar to those observed in planar lipid bilayer measurements^48^. A similar experiment was also done with lysozyme (Supplementary Figure 5).

Electrical capacitance was used to confirm the formation of a lipid bilayer between the aqueous droplet and the hydrogel^42^,^45^,^46^. Using a syringe, an aqueous droplet was formed at the end of a microneedle inside the oil volume, and transferred to the end of an insulated Ag/AgCl electrode pierced through the hydrogel. Another insulated Ag/AgCl electrode was inserted into the hydrogel block (Supplementary Figure 3). The droplet was brought into contact with the hydrogel surface with micrometer precision using a micromanipulator (Fig. 4a). Bilayer formation was deduced by an increase in the capacitance (Fig. 4b). Using a specific capacitance of 0.65 μF cm^−2^ and a maximum measured bilayer capacitance of ~5000** **pF, a maximum contact area of ~0.8 mm^2^ was estimated between the droplet and the hydrogel surface^47^. The bilayers formed were stable for at least 1 h at +50 mV (Supplementary Figure 4). By manipulating the electrode connected to the micromanipulator, the bilayer area could be changed as shown by a change in the electrical capacitance (Supplementary Figure 4).

### Functionalizing encapsulated aqueous droplet-hydrogel bilayers by means of protein nanopores

Previously, it was shown that bilayers formed between two droplets (convex - convex contact)^45^, between a droplet and a hydrogel (convex – flat contact)^44^, or two hydrogel pieces (flat – flat, convex – flat, convex - convex contacts)^42^, are capable of hosting protein nanopores and ion channels^46^. Here, the encapsulated bilayer was formed between a convex aqueous droplet and a concave hydrogel surface.

Owing to its evolving importance as a nanopore sensor for proteins^48^ and DNA^49^, and the possibility to engineer its size and properties^50^, we chose ClyA as the model nanopore to test our system. The encapsulated bilayer could be functionalized with ClyA pores by incorporating the oligomeric pore inside the aqueous droplet (Fig. 4c). The conductance of ClyA inserted in the hydrogel-encapsulated proto-organelle bilayer (1.9 ± 0.3 nS; *n = *5) was similar to the ClyA conductance measured in a conventional planar lipid bilayer (1.79 ± 0.04 nS; *n = *3) (Fig. 4d). Thus, the geometry of the contact surfaces did not affect the integrity of the bilayer and the assembly of the membrane pores in the lipid bilayer.

The stability of the ClyA pore was further ascertained by monitoring the binding kinetics of human thrombin (huThr, 37 kDa) from the *cis* entrance of the pore^48^. An aqueous droplet with huThr and ClyA was transferred onto an Ag/AgCl electrode in an oil chamber inside hydrogel. Bilayer formation between the droplet and the hydrogel and subsequent nanopore insertion were monitored electrically. Transient closure of a single ClyA pore, denoting the entry and exit of huThr, was measured (Fig. 4f). huThr inside ClyA nanopores produced two current levels. At −35 mV, the I_*RES*_ of level 1 is 53.2% ± 1.6% (*N = *3, *n = *263; where *N* is the number of experiments and *n* is the number of events) and the I_*RES*_ of level 2 is 21.5% ± 2% (*n = *265). The measured values were in good agreement with those determined in planar lipid bilayers^48^. Transient blocking events were also observed when a smaller protein such as lysozyme (15 kDa) was used instead of huThr (Supplementary Figure 5). The dwell time (*τ*_d_) of lysozyme blockades was 0.5 ± 0.2 ms (*N = *3, *n = *670), which is similar to that measured in ClyA inserted in a planar lipid bilayer (0.42 ms).

The ClyA pores inserted in the encapsulated aqueous-hydrogel bilayer were also capable of transporting the small molecule pyranine. A multi-compartment hydrogel was formed by encapsulating two oil volumes inside the same hydrogel piece. Aqueous droplets containing pyranine (one with and one without ClyA) were injected into each of the oil chambers (Supplementary Figure 6a). The droplet with ClyA showed a gradual decrease in fluorescence over 23 h, whereas the one without ClyA did not show a decrease in fluorescence and retained the dye.

### Communication between hydrogel-encapsulated aqueous droplets

Next, we demonstrated communication between aqueous droplets when encapsulated either in the same oil compartment or in different oil compartments inside the hydrogel. A linear array of 3 aqueous droplets in oil encapsulated in hydrogel was formed; the middle droplet contained pyranine, one terminal droplet contained ClyA and one did not. Pyranine diffusion was observed only across the bilayer between the droplet with pyranine to the droplet containing ClyA (Supplementary Figure 6b).

**Figure 5:**
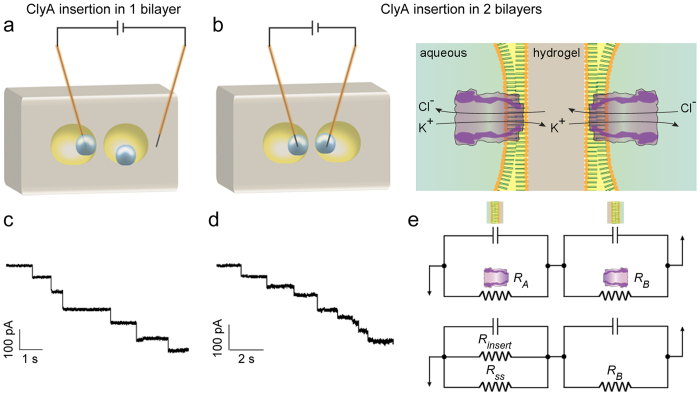
Electrical communication between proto-organelles of a protocell. Owing to the firm enclosure of oil drops in a hydrogel, it was possible to reduce the distance between the oil drops to a thin layer of agarose (100–200 μm). Two aqueous droplets, one in each oil chamber, were controlled using micromanipulators to form contiguous bilayers. The formation of the two bilayers was confirmed by electrical capacitance measurements (Supplementary Figure 7), and also by the insertion of ClyA pores. (**a**,**c**) First, an electrical connection was formed only between one aqueous droplet containing ClyA (1–10 ng mL^−1^) and the hydrogel by inserting two insulated electrodes, one in the aqueous droplet and one in the hydrogel. The unitary conductance of ClyA was 1.9 ± 0.3 nS. (**b**,**d**) Next, in the same experimental set-up, an electrical connection was established between the two droplets (both containing 1–10 ng mL^−1^ ClyA) by inserting an electrode in each droplet. As expected in the case of two bilayers in series, the amplitude of individual current steps decreased with ClyA insertions. (**e**) The top panel shows the electrical model where single ClyA pores (resistors) are inserted in two adjacent bilayers (capacitors). The bottom panel shows the insertion of an additional ClyA pore in the left bilayer. The step-sizes of individual insertions can be modelled (Supplementary Note 1).

Oil encapsulation inside hydrogel affords the possibility of controlled compartmentalization and the formation of abutting lipid bilayers. Aqueous droplet-hydrogel bilayers were formed in adjacent oil compartments (Fig. 5, Supplementary Figure 3e). The simultaneous formation of two bilayers was determined by measuring an increase in electrical capacitance (Supplementary Figure 7). Step-wise ClyA insertion was observed in both of the juxtaposed bilayers. The apparent unitary conductance decreased with each insertion as expected for resistors in series (Fig. 5, see Supplementary Note 1 for electrical model).

### A nested system: adding an aqueous layer between the hydrogel and the oil

**Figure 6:**
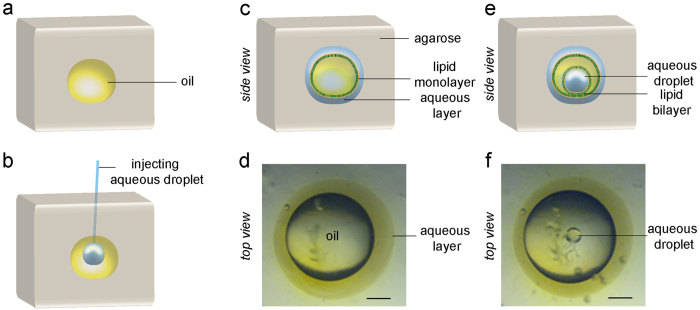
A multi-layered hydrogel encapsulated system. The aqueous droplet-hydrogel module was further engineered by introducing an aqueous layer between the oil and hydrogel compartments. (**a**) An oil chamber was formed inside the hydrogel. (**b**,**c**) A large aqueous volume (~500 μL) was injected into the oil. Instead of forming a stable bilayer at the oil/hydrogel interface the aqueous volume engulfed the oil. (**d**) Image of the system depicted in (**c**). (**e**) A small aqueous droplet was injected inside the oil (proto-organelle) such that the aqueous droplet was stabilized by a bilayer at the oil/aqueous layer interface. (**f**) Image of the system depicted in (**e**). The aqueous layer between the oil and hydrogel was stable for at least 21 h (Supplementary Figure 8). Scale bars, 1 mm.

To demonstrate the versatility of the present system, an aqueous layer was introduced between the hydrogel matrix and the oil (Fig. 6). The oil-lipid mix was encapsulated as before at the hydrogel gelling transition temperature. Instead of injecting a small aqueous volume (~50 nL), ≥500 μL were injected into the oil forcing the aqueous phase to engulf the oil drop and form an intervening layer between the hydrogel and the oil. A small aqueous droplet could still be injected inside the oil drop to form a stable bilayer at the oil – aqueous layer interface, all encapsulated in hydrogel (Fig. 6).

## Discussion

Liposomes^34^,^51^ and polymersomes^52^ have been used to form multiple compartments encapsulated in hydrogel as potential cell mimics, drug delivery vehicles and biosensors. However, these nanometer-sized containers are not amenable to spatial control within the structures, nor is it possible to achieve a precise control of the number of compartments inside the hydrogel. A defined number of aqueous compartments would be an important feature of a protocell for a controlled functional output. Recently, encapsulation of aqueous droplets inside alginate shells using a microfluidic platform was demonstrated^53^. The system, similar to the one presented here, was stable in air, oil and aqueous media. Although the microfluidic approach is useful for encapsulating specific numbers of aqueous droplets inside a single oil volume, the formation of multiple oil compartments may be challenging. The protocell model presented in this report is a simple manual assembly of aqueous compartment(s) in oil inside a hydrogel matrix. The solid support provided by the hydrogel and the ease of manual construction of the module allows precise control over the number of aqueous and oil chambers inside the hydrogel. A hierarchical architecture where the number of compartments can be controlled is especially important for engineering linear input-output systems, which can be developed into more complex systems with non-linear outputs^54^.

Importantly, in both, the vesicular^55^,^56^ and the droplet-based multi-compartment systems^22^, any two compartments share one bilayer. This situation is unlike that in a biological cell (or a tissue) where individual organelles (or cells) are demarcated by separate lipid bilayer membranes. The protocell based on the hydrogel module makes it possible to include proto-organelles with separate bilayers (Fig. 2), pivotal towards demonstrating inter-organelle electrical communication (Fig. 5). The next milestone would be to demonstrate a direct connection between two adjacent bilayers for mass transfer^57^,^58^. In the future, it would also be worth engineering the droplet-hydrogel platform to enable active exchange of nutrients and waste materials between the droplets encapsulated in the hydrogel or between the droplets and the hydrogel^56^,^59^.

Owing to its basic constituents: hydrogel, oil, water, lipids, the hydrogel-encapsulated lipid bilayer protocell has the capacity to be functionally upgraded by means of physical, chemical, and biological engineering. Expanding the chemical and physical repertoire of the platform (e.g., by using different polymers, oils and lipids) will enable the construction of units capable of responding to biological^60^, chemical^61^ or physical cues^62^,^63^. In light of the results presented in this report, the successful reconstitution of ion channels in droplet bilayers^64^, and the similar behavior of mechanosensitive channels in cells and in droplet bilayers^65^, the use of the droplet-hydrogel system can be considered as a significant step toward developing a cell mimic. Because lipid bilayers can form on a hydrated support cushion^66^, the inclusion of an intervening aqueous layer between the hydrogel and the aqueous droplet in oil (Fig. 6) demonstrates the possibility of forming concentric lipid bilayers akin to those in organelles with double bilayers (mitochondrion, nucleus) in biological cells.

Similar to capsosome-, polymersome- and vesosome-based multi-compartment structures^67^ which have been proposed as drug vehicles^52^,^68^, the hydrogel system presented here might be used, for example, to package incompatible drugs, or prodrugs and activators in different compartments. The use of hydrogel, lipids and oil might be exploited to precisely tune the physical properties of the unit thereby permitting controlled release of the active ingredients (e.g., siRNA, nanoparticles, chemotherapeutics) at the site of interest (e.g., by surgical implantation of the hydrogel module)^69^.

The right type of hydrogel could provide a suitable milieu for growing cells enabling the synthetic compartments to be connected with biological cells or tissues^70^. Cells can be encapsulated in 3D hydrogel matrices^71^ for interfacing cell signaling with *in vitro* signaling^72^, tissue engineering^73^, regenerative therapies^74^ or for organoid development^75^,^76^. Crucially, hydrogels can provide tailored 3D niches for stem cell differentiation^77^ and proliferation^78^. High-throughput automated systems (e.g., microfluidics^53^, 3D printing^79^) would be instrumental in miniaturizing the droplet-hydrogel unit for the aforementioned goals, implantable therapeutic agents and biosensors.

## Methods

### Liposome preparation

25 mg of 1, 2- diphytanoyl-sn-glycero-3-phosphocholine (DPhPC) (Avanti Polar Lipids, USA) was dissolved in 1 mL pentane (Chem-Lab, CL00-1614) in a glass vial. The lipid solution was dried under a stream of filtered nitrogen (N_2_) to form a uniform lipid film on the glass surface. The dried lipid was suspended in a buffer (150 mM NaCl, 10 mM Tris-HCl, pH 7.5), followed by sonication and extrusion (11 times) through a filter (pore size 0.2 μm) to give unilamellar liposomes. The liposome solution was diluted to a final concentration of 5 mg mL^−1^ DPhPC in 150 mM NaCl, 10 mM Tris-HCl, pH 7.5. Liposomes from porcine brain lipid extract (Avanti Polar Lipids, USA) were made in the same way.

### Oil composition

Lipids (DPhPC or porcine brain lipid extract) dissolved in pentane at 10 mg mL^−1^ were evaporated using a stream of filtered N_2_. The dried lipid film was re-solubilized in a 1:1 (v/v) mixture of silicone oil (AR 20, Sigma-Aldrich, 10836) and hexadecane (Sigma-Aldrich, H6703). For electrical experiments hexadecane and silicone oil were mixed in a ratio of 3:1 (v/v).

### Hydrogel composition

1% w/v low-gelling temperature agarose (Sigma-Aldrich, A9414) was dissolved by heating in the same buffer used to make liposomes (150 mM NaCl, 10 mM Tris-HCl, pH 7.5).

### Hydrogel formation and enclosing single and multiple oil compartments in the hydrogel

Agarose gel was melted by heating to 80 °C and poured into a 10 mm × 35 mm plastic cuvette (Sigma-Aldrich, BRAND UV cuvette, Z637157). The temperature was monitored every 30 s by means of a hand-held thermistor. At the sol-gel transition temperature of the agarose (27 ± 1 °C), an oil/lipid mixture was injected using a Hamilton syringe to form an oil drop inside the hydrogel. Multiple compartments of oil inside the hydrogel were formed in the same manner. After injecting one oil drop inside the hydrogel, the syringe was withdrawn and immediately re-inserted to inject a second oil drop very close to the first one. Up to 10 oil drops (50–200 μL) could be injected into a single hydrogel block. If injected at a higher temperature, when the agarose was in the sol state, the oil escaped. At a lower temperature, when the agarose had already gelled, it was difficult for the oil to displace the agarose by an equivalent volume. The hydrogel piece with the oil drop was allowed to cool to room temperature (22 ± 1 °C). The hydrogel block was then removed from the plastic cuvette by gently pushing and placed in a Petri dish under oil to prevent drying of the agarose.

### Encapsulating aqueous droplets in the oil/hydrogel

A 1 mL plastic syringe (BD LUER-LOK) filled with DPhPC liposomes (2–5 mg mL^−1^) was attached to a ~2 cm long MICROFIL needle (CMF90UxxL, 36 gauge, 20 μm inner diameter, 90 μm outer diameter; World Precision Instruments). The needle was inserted into the encapsulated oil drop by piercing the solidified hydrogel encasing (22 ± 1 °C), and was held on the upper edge of the oil drop taking care that the needle end did not touch the hydrogel. By a slight push of the piston, the liposome solution was expelled to form a small aqueous droplet (100–400 μm in diameter). A quick withdrawal of the MICROFIL needle from the oil allowed the aqueous droplet to separate from the needle and slowly fall to the bottom of the oil drop. The small holes created in the hydrogel by piercing with the needle became resealed. Keeping the hydrogel in oil also prevented the oil oozing out of the hydrogel by maintaining a flow equilibrium while the holes became resealed or if the holes did not properly reseal.

### Aqueous droplet stability measurements

Many sets of agarose blocks each with encapsulated oil mixture (7.5 mg mL^−1^ DPhPC) were made. Assemblies of aqueous droplets (2, 4, 6, 8 droplets) containing liposomes (5 mg mL^−1^ DPhPC in 150 mM NaCl, 10 mM Tris-HCl, pH 7.5) were injected into each oil drop and the constructs were placed in an incubator at 29 °C. To monitor the stability at 37 °C, aqueous droplets (1, 2, 4, 6 droplets) containing liposomes (10 mg mL^−1^ DPhPC) were placed in the incubator at 29 °C and the temperature raised to 37 °C over 2 h. The stability criterion was the time until one of the aqueous droplets fused with the hydrogel or until two droplets fused with each other to make a bigger droplet. The experiment was monitored every 12 h for up to 4 d.

### Expression of ClyA pores

A engineered ClyA containing five mutations, S87C, L99Q, E103G, F166Y and K294R, was used^80^. Monomers containing a C-terminal oligo-histidine tag were expressed in *E. coli* BL21 cells and the soluble fraction purified using Ni-NTA affinity chromatography. Oligomerization of ClyA dodecamers was triggered by the addition of 0.5% w/v β-dodecylmaltoside (DDM, GLYCON Biochemical, GmbH) and incubation for 15 min at 37 °C. Different oligomeric states and monomeric ClyA were separated by blue native polyacrylamide gel electrophoresis using 4–20% polyacrylamide gels (BN-PAGE, Bio-Rad). The band corresponding to dodecamer ClyA was excised from the gel and placed in 150 mM NaCl, 15 mM Tris.HCl, pH 7.5 supplemented with 0.2% w/v DDM and 10 mM EDTA to allow diffusion of the protein out of the gel^80^. The resulting oligomeric ClyA was stored at 4 °C for up to 3 weeks.

### Bilayer formation and capacitance measurement

A small piece of the insulating layer was removed from one end of PFA (perfluoroalkoxy)-insulated silver wire (200 μm diameter, A-M systems, USA). Ag/AgCl electrodes were made by immersing the exposed end of the wire in a sodium hypochlorite solution for at least 1 h. 3% w/v melted agarose was dabbed at the end of the electrodes to prevent slippage out of the aqueous droplets^45^. Current was measured across the bilayer formed between the encapsulated aqueous droplet (5 mg mL^−1^ DPhPC in 150 mM NaCl, 10 mM Tris-HCl, pH 7.5) and the hydrogel by inserting an Ag/AgCl electrode (*cis*) attached to the ground terminal of the headstage into the aqueous droplet (Axon Instruments, USA). Another Ag/AgCl electrode (*trans*), connected to the active terminal of the headstage, was inserted in the hydrogel. A micromanipulator was used to control bilayer formation by moving the aqueous droplet to touch the inner wall of the hydrogel.

To measure the bilayer capacitance between two contiguous aqueous drops, two oil drops were injected into an agarose block as described above. The *cis* electrode, attached to the ground end of the patch-clamp headstage, was inserted into one oil drop. The *trans* Ag/AgCl electrode was inserted into the other oil drop. Aqueous droplets (5 mg mL^−1^ DPhPC in 150 mM NaCl, 10 mM Tris-HCl, pH 7.5) were injected directly onto the electrodes using a micro needle. The distance between the aqueous droplets was adjusted by using micromanipulators to form bilayers with the hydrogel.

### Single channel electrical measurement of ClyA in single and double bilayers

For electrical recording ~1–10 ng oligomeric ClyA was added to an aqueous droplet (5 mg mL^−1^ DPhPC in 150 mM NaCl, 10 mM Tris-HCl, pH 7.5) connected to the *cis* electrode. The *trans* electrode was inserted into the hydrogel to form a single droplet-hydrogel bilayer (Fig. 4). To form adjacent bilayers (Fig. 5), the *trans* electrode was connected to a ClyA containing aqueous droplet (5 mg mL^−1^ DPhPC in 150 mM NaCl, 10 mM Tris-HCl, pH 7.5).

### Single channel electrical measurements of thrombin and lysozyme blocking of ClyA

Stock solutions of human thrombin (huThr) were prepared by dissolving the lyophilized protein (Sigma-Aldrich, T6884) in MILLI-Q water to a concentration of 0.2 NIH units μL^−1^. The solution was aliquoted and stored at −20 °C. The molar concentration of HT was calculated from its unit concentration, with 1 NIH unit mL^−1^ = 10 nM. huThr (10 nM) was added to an aqueous droplet containing ClyA (1–10 ng), which was connected to the *cis* electrode. The *trans* electrode was inserted in the hydrogel.

For the lysozyme experiment, 3 μg of lysozyme (chicken egg white, Sigma L6876) was dissolved in 500 μL of liposomes (5 mg mL^−1^ DPhPC in 75 mM NaCl, 7.5 mM Tris-HCl, pH 7.5). The final concentration of lysozyme was 400 nM. The configuration of the *cis* and *trans* electrodes was the same as in the experiment with huThr. Electrical signals were amplified using an Axopatch 200B patch clamp amplifier (Axon Instruments) and digitized with a Digidata 1440A/D converter (Axon Instruments). Data were recorded using Clampex 10.4 software (Molecular Devices) and subsequent analysis was carried out using Clampfit software (Molecular Devices). The signal was filtered with a 2 kHz low-pass Bessel filter and sampled at 10 kHz. All electrical measurements were conducted at 25 °C.

### Optical measurements

For optical measurements involving pyranine in aqueous droplets, ClyA monomers (not labeled with any dye) were added to 0.2% w/v DDM in 150 mM NaCl, 15 mM Tris.HCl pH 7.5. The ClyA oligomer so formed (20–80 μg mL^−1^) was mixed with DPhPC liposomes, and aqueous droplets were injected into oil encapsulated in hydrogel. The hydrogel was kept immersed in oil to prevent drying during the course of the experiment.

A fluorescence stereo microscope (Leica M165 FC) was used for all optical experiments. Fluorescence images were acquired using a GFP filter (10 447 408 Filter Set ET GFP LP - M205FA/M165FC). ImageJ software (Fiji) was used to analyze and process recorded images. In the experiments, the diffusion of pyranine was monitored.

## Additional Information

**How to cite this article:** Bayoumi, M. *et al*. Multi-compartment encapsulation of communicating droplets and droplet networks in hydrogel as a model for artificial cells. *Sci. Rep.* **7**, 45167; doi: 10.1038/srep45167 (2017).

**Publisher's note:** Springer Nature remains neutral with regard to jurisdictional claims in published maps and institutional affiliations.

## Supplementary Material

Supplementary Information

Supplementary Movie 1
