# Supplementary material for: Multi-compartment encapsulation of communicating droplets and droplet networks in hydrogel as a model for artificial cells
Source: Sci Rep. 2017 Apr 3;7:45167. doi: 10.1038/srep45167 (PMC5377250; doi:10.1038/srep45167)
Supplement: Supplementary Information [file srep45167-s1.pdf]

Multi-compartment encapsulation of communicating droplets and droplet networks in hydrogel as a model for artificial cells

Mariam Bayoumi [1], Hagan Bayley [2], Giovanni Maglia [1,3], K. Tanuj Sapra [4]

[1] Department of Chemistry, KU Leuven, Celestijnenlaan 200G, 3001 Leuven, Belgium

[2] Chemistry Research Laboratory, 12 Mansfield Road, Oxford OX1 3TA, United Kingdom

[3] Groningen Biomolecular Sciences and Biotechnology Institute, University of Groningen, Nijenborgh 7, 9747 AG Groningen, The Netherlands

[4] Department of Biochemistry, University of Zurich, Winterthurerstrasse 190, 8057 Zurich, Switzerland

Address correspondence to:

Giovanni Maglia
Email: g.maglia@rug.nl

or

K. Tanuj Sapra
Email: k.sapra@bioc.uzh.ch

Supplementary Figures

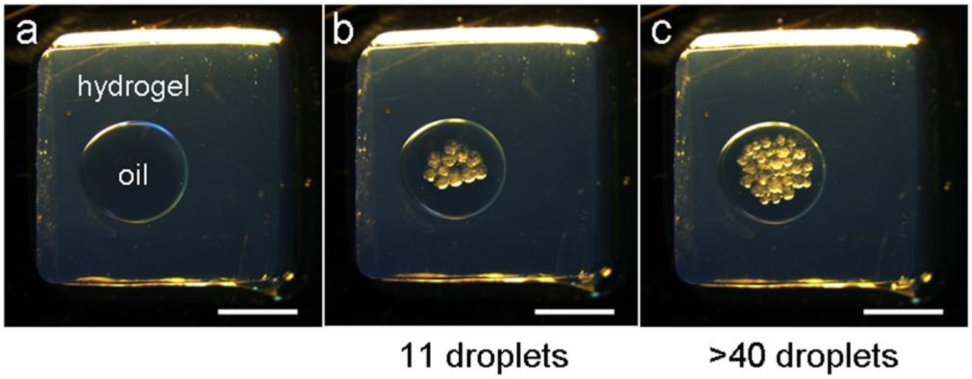

Supplementary Figure 1. Encapsulation of aqueous droplets in hydrogel. (**a**) An oil drop encapsulated in hydrogel. (**b, c**) Increasing number of aqueous droplets were injected in the oil. Adding more aqueous droplets to the oil did not show a noticeable change in the oil volume. Scale bars, 5 mm.

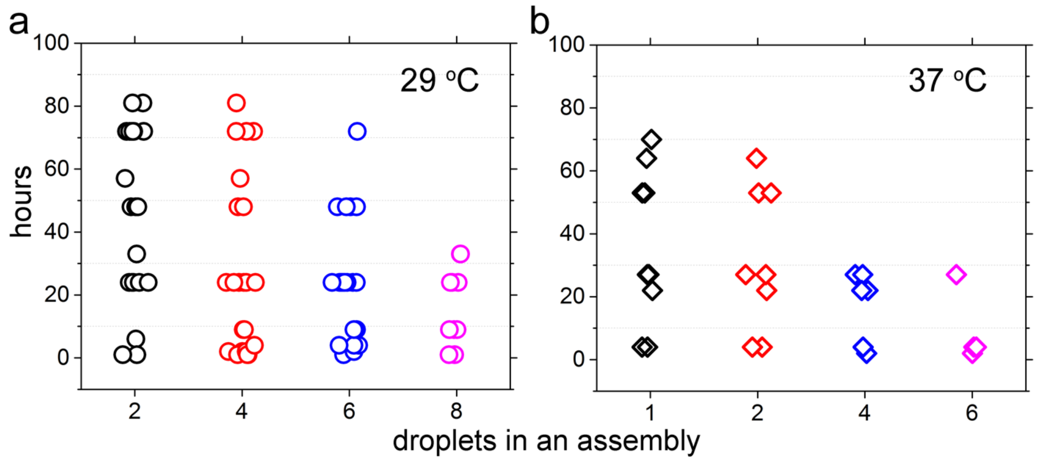

Supplementary Figure 2. Stability of hydrogel-encapsulated aqueous droplets in different assemblies. (**a**) Assemblies with 2, 4, 6 and 8 aqueous droplets (400 μm diameter, containing 5 mg mL^{-1} DPhPC) were injected into an oil / lipid mixture (7.5 mg mL^{-1} DPhPC) encapsulated in different hydrogel blocks and their stability monitored at 29 °C. (**b**) Similarly, aqueous droplets (100 – 400 μm diameter, containing 10 mg mL^{-1} DPhPC) were injected in an oil / lipid mixture (7.5 mg mL^{-1} DPhPC) were encapsulated in different hydrogel blocks and their stability monitored at 37 °C. The hydrogel pieces were kept immersed in oil during this time to prevent drying and shrinkage of the hydrogel. The plot shows the time taken for the first aqueous droplet to fuse with the hydrogel or with another aqueous droplet. The remaining droplets were still intact. As shown, the stability of the first droplet ranged between 1 h up to 81 h. With increasing size of the assemblies the maximal stability of the first droplet decreased from ~80 h to ~30 h at both 29 °C and 37 °C.

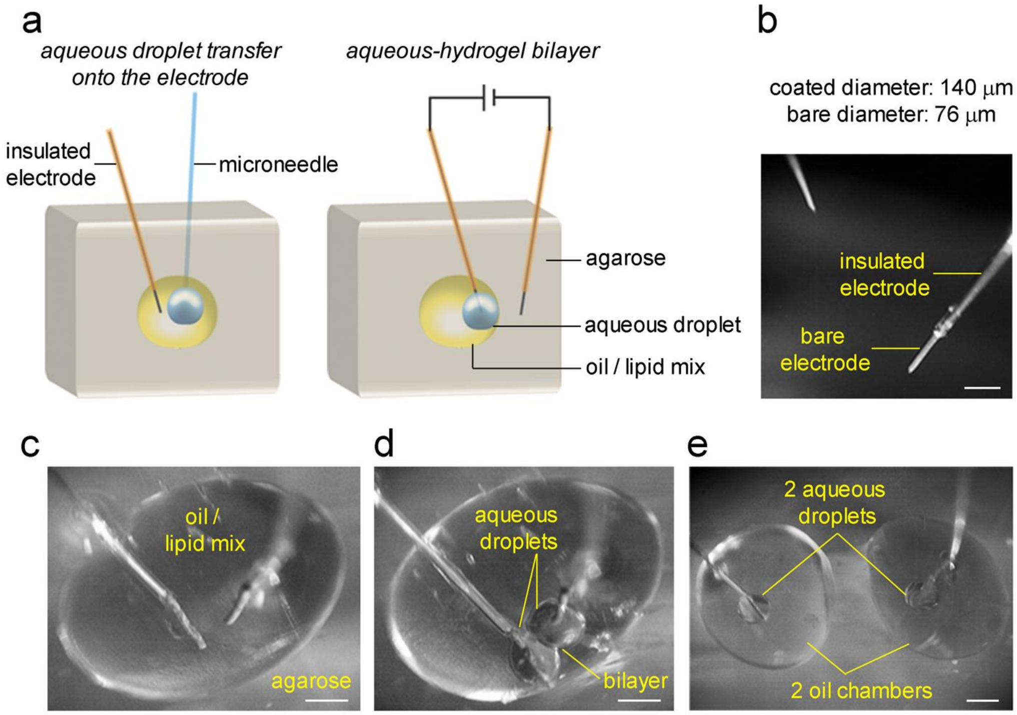

Supplementary Figure 3. Set-up for the electrical measurement of encapsulated droplet-hydrogel bilayers. (**a**) Diagram showing the formation of a droplet-hydrogel bilayer for electrical recording. An oil chamber was formed by injecting a specific volume (10 – 100 µL) of oil into the hydrogel (**Figure 2**). An insulated electrode was then inserted inside the oil, and an aqueous droplet transferred onto the electrode using a microneedle. Another insulated electrode was inserted in the hydrogel and the electrodes were connected to an amplifier. (**b**) Photograph of an insulated electrode with dimensions. Besides droplet-hydrogel bilayers, the system could also be used to form droplet-interface bilayers [1]. (**c**) Two electrodes inserted inside the same oil volume; (**d**) aqueous droplets transferred onto the electrodes and brought together using a micromanipulator to form a droplet interface bilayer. (**e**) Droplet-hydrogel bilayers could be formed in two closely spaced oil chambers to give two bilayers. Stable bilayers with either DPhPC or porcine brain total lipid extract could be formed. Scale bars, (**b – e**) 300 µm.

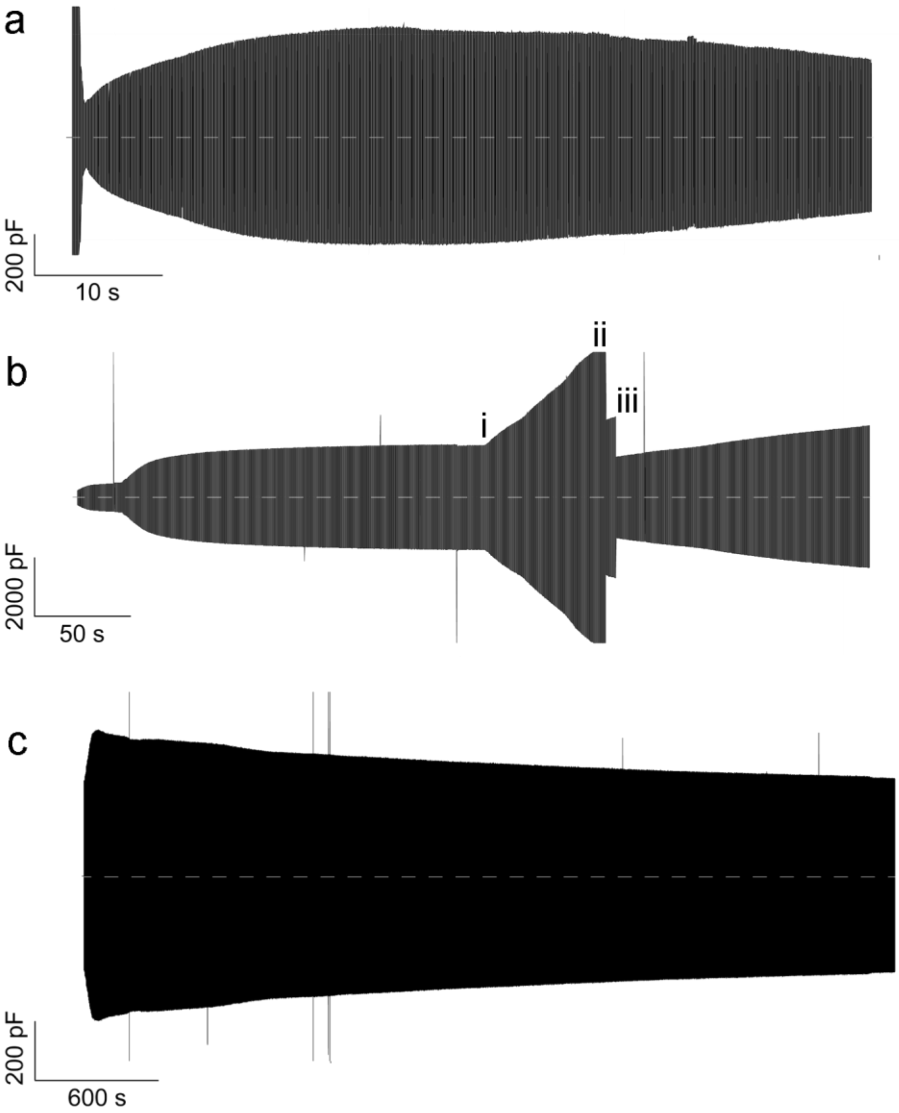

Supplementary Figure 4. Stable droplet-hydrogel bilayer formation. (**a**) Capacitance increase indicating the formation of a bilayer between an aqueous droplet and hydrogel at +50 mV. (**b**) The bilayer area could be increased (i) or decreased (ii, iii) by pushing or pulling the encapsulated aqueous droplet onto or against the hydrogel wall, respectively. (**c**) Capacitance recording of a bilayer stable for >1 h under an applied potential of +50 mV.

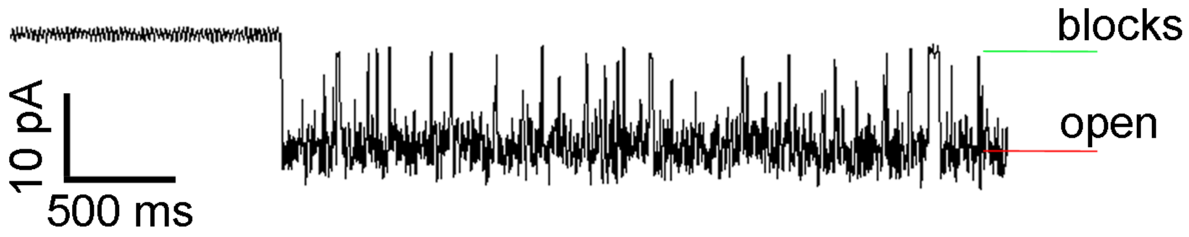

Supplementary Figure 5. Transient blocking of ClyA with lysozyme. Besides huThr (37 kDa), lysozyme (15 kDa) was shown to transiently interact with the ClyA pores inserted in the encapsulated droplet-hydrogel bilayer.

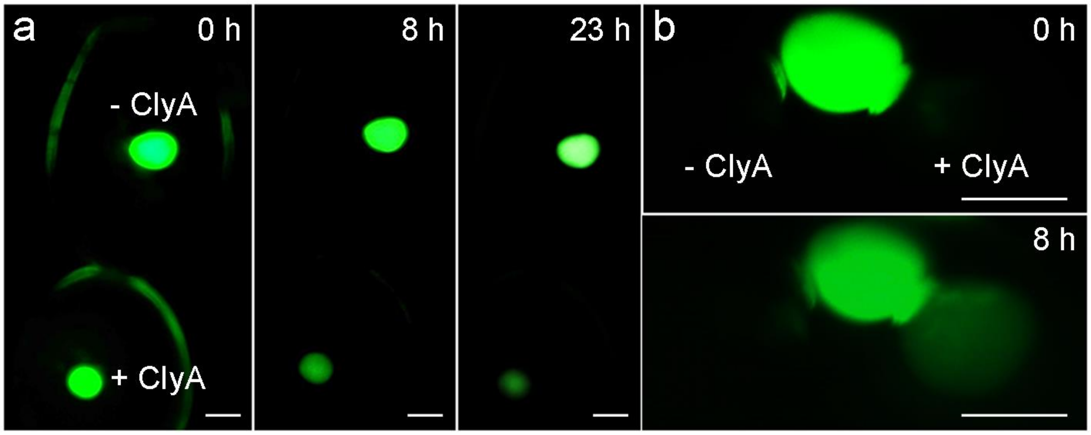

Supplementary Figure 6. Functional proto-organelle bilayers. (**a**) Two droplets containing pyranine (10 mM in 150 mM NaCl, 10 mM Tris HCl, pH 7.5) (solid green spheres) were injected in two separate oil drops (green outline) encapsulated in a hydrogel (immersed in oil to prevent drying). In one droplet (labeled +ClyA) ClyA (60 μg mL^{-1}) was added but not in the other (-ClyA). Insertion of ClyA pores (not labeled) in the droplet-hydrogel bilayer enabled diffusion of pyranine outside the droplet (into the hydrogel) as seen by a decrease in the green intensity. No decrease in intensity was observed in the droplet without ClyA over the same duration (~23 h). (**b**) A similar experiment was performed with a linear assembly of 3 droplets enclosed in an oil chamber in an agarose block. The middle droplet with pyranine (green) did not contain ClyA. The droplet on the left was also without ClyA (-ClyA), whereas ClyA was incorporated in the droplet on the right (+ClyA, 40 μg mL^{-1}). The interface with +ClyA droplet allowed pyranine transport turning the droplet green over 8 h. The –ClyA droplet did not show an increase in green intensity during the same time. Scale bars, 200 μm.

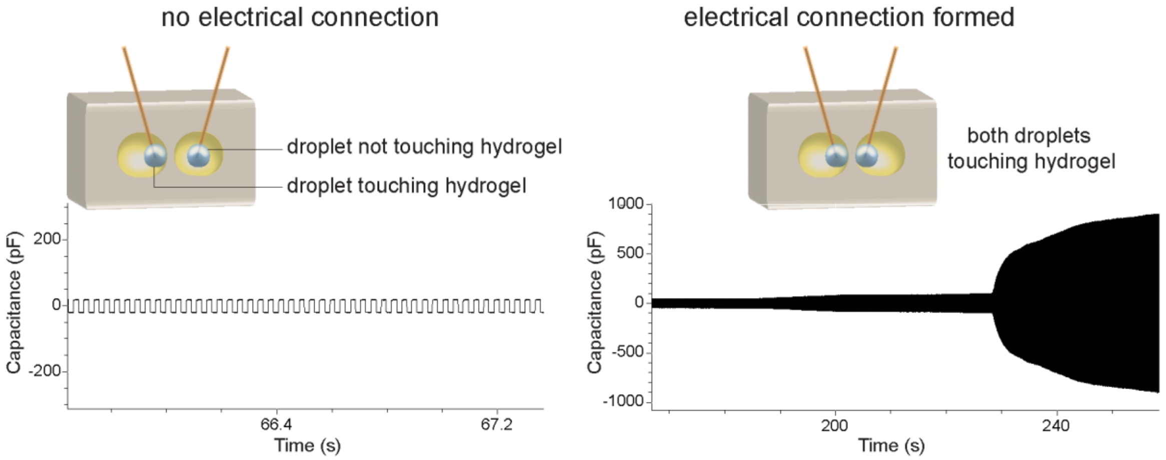

Supplementary Figure 7. Simultaneous formation of adjacent bilayers. Two aqueous droplets were transferred onto insulated Ag / AgCl electrodes inserted into two oil chambers next to each other, encapsulated in hydrogel. (**a**) Only one droplet was used to form a bilayer by touching the hydrogel using a micromanipulator. No increase in the electrical capacitance was observed. (**b**) Upon touching the other aqueous droplet to the hydrogel, the circuit was complete and an increase in the electrical capacitance was observed indicating the formation of two bilayers.

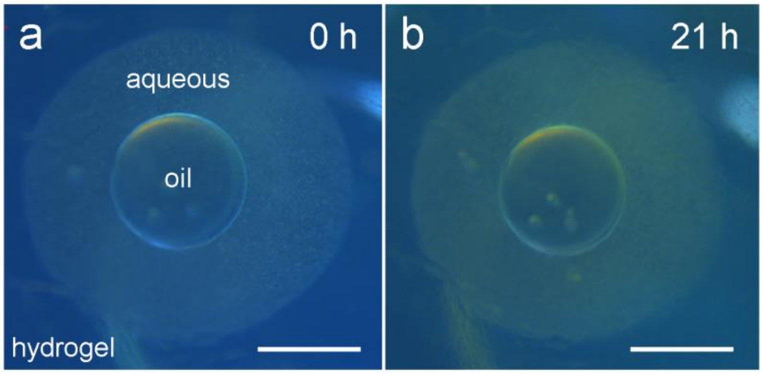

Supplementary Figure 8. Stability of aqueous layer between oil and hydrogel. (**a, b**) The aqueous layer between the oil and the hydrogel did not show a drastic change in its diameter (measured from the center of the oil droplet) over 21 h. Scale bars, 2 mm.

Supplementary Movie 1. Aqueous droplet injection inside an oil drop encapsulated in hydrogel. The real time movie shows sequential manual injection of aqueous droplets using a syringe needle. At the start of the movie, an aqueous droplet sits stably in an oil volume inside a hydrogel block (not seen in the movie). Small volumes of aqueous droplets are then injected which fuse with the big droplet without affecting its integrity.

Supplementary Note 1. Electrical model of nanopore insertion in adjacent lipid bilayers encapsulated in hydrogel.

Bilayer formation and nanopore insertion in the hydrogel-enclosed droplet system can be treated as the formation and modification of an electrical circuit in which droplet-hydrogel bilayers are capacitors, ClyA pores are resistors, and the hydrogel between the two electrical circuits is considered as a conductive junction. In the case of a single droplet-hydrogel bilayer the insertion of each pore is characterized by a current step of the same magnitude since the current results from single resistors (pores) inserting sequentially into the bilayer. In the case of two bilayers placed adjacent to each other, the current steps are variable. We only consider the case of two adjacent bilayers in a hydrogel.

Let's say two adjacent bilayers are formed by two aqueous droplets, *A* and *B*, with the hydrogel. The resistance of the nanopores inserted in the two droplet-hydrogel bilayers is denoted as R_A and R_B, respectively (**Figure 5e**). Because the two bilayers are connected through the hydrogel, we consider them as two electrical circuits connected in series. Applying rules from the Kirchhoff's Circuit Law and Ohm's Law we derive basic equations for the droplet-hydrogel adjacent bilayers system.

The net resistance, R_t, is the total resistance of the two series circuits (bilayers) with resistors R_A and R_B,

$$R_t = R_A + R_B \tag{1}$$

Pore insertion in the adjacent bilayer system can be characterized by two types of current; steady-state current, I_{ss}, and insertion current, I_{insert} (t). I_{ss} is the current after pore insertion, whereas I_{insert} (t) is the current at the moment of pore insertion.

The steady-state current through the pores in adjacent bilayers is given simply by Ohm's Law, $I = V/R$. Therefore, I_{ss}, after the insertion of a pore under an applied voltage, V_{cc}, is,

$$I_{ss} = \frac{V_{cc}}{R_A + R_B} \tag{2}$$

From equation (2) we can expect that I_{ss} resulting from the two bilayer system is less than the one from one bilayer with the same type of pores (resistors). The average I_{ss} of single pores in the one bilayer system is -67 ± 11 pA at -35mV (**Figure 5c**). This is in agreement with the conductance of ClyA nanopores [2]. In the case of two adjacent bilayers the average I_{ss} is -29.5 ± 34 pA at -35 mV (**Figure 5d**).

In order to obtain a model which describes the current during the continuous insertion of pores in the two adjacent bilayer system, i.e., I_{insert} (t), more resistors have to be added in the electrical circuits. Equation (3) describes the current at the moment of insertion (I_{insert} (t)) [3],

$$I_{insert}\,(t) = \propto + \beta\exp(-\gamma t) \tag{3}$$

where,

α steady-state net current through two adjacent bilayers when an additional pore inserts

β initial current deviation from α immediately after a pore insertion

γ inverse of exponential decay time constant for pore insertion event

The numerical solution of equation (3) gives 3 possibilities for β; = 0, > 0 or < 0. These values depend on the ratio of the number of pores inserted in each bilayer. In our experiments we could observe the 3 different possibilities denoting the insertion of ClyA in adjacent bilayers **(Figure SN1**).

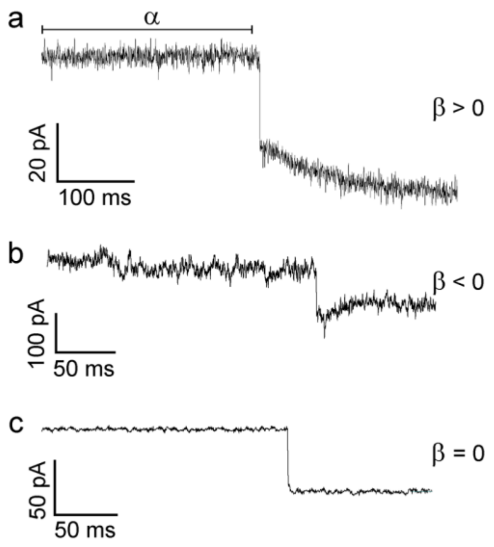

Figure SN1. Types of current traces observed during pore insertion in the two bilayer system.

From equations (2) and (3) we can determine the insertion configurations of pores in bilayers *A* and *B*. From equation (3), if β = 0, I_{insert} (t) is equal to α, i.e., it is equal to the steady-state current. Thus, we can use equation (2) to determine the number of pore insertion and where each new pore inserts in the two bilayers. However, to be able to use equation (2) we have to determine the resistance of ClyA nanopores.

The conductance of a ClyA nanopores at ±35 mV is 2.03 nS and 1.79 nS respectively [2]. Thus, the resistance values of the ClyA pore at ±35 mV are 0.55 GΩ and 0.49 GΩ, respectively. This difference in the resistance at the same voltage is due to the rectification of the nanopore. In the adjacent bilayer system, ClyA nanopores inserted in bilayer *B* will have a different orientation than the pores inserted in bilayer *A*. Therefore the resistances of the pores in the two bilayers will have different values at the same voltage due to the rectification properties of ClyA. For example, at -35 mV, R_{SS} = 0.55 nΩ in case of bilayer *A* (connected to the ground electrode) and R_B = 0.49 nΩ for the pore in bilayer *B* (connected to the active electrode).

If more than one pore is inserted in any bilayer of the two adjacent bilayers, then those pores are resistors connected in parallel. If we assume that a new pore with resistance R_{insert} is inserted in bilayer *A*, and R_{ss} is the resistance of bilayer *A* before the insertion of the new pore, then the total resistance, R_A, of bilayer A is,

$$R_A = R_{insert} \parallel R_{ss} \qquad R_A = \frac{R_{insert} \times R_{ss}}{R_{insert} + R_{ss}} \tag{4}$$

The value of the current steps denotes the configuration of the pores in the bilayer. The step-size in **Figure SN2a** denotes a pore in configuration (1A,1B), i.e., a single pore is inserted in bilayer *A* and another pore in bilayer *B* . The change of 33.9 pA upon pore insertion observed experimentally agrees with the calculated value of 33.6 pA (1A,1B) from equation (2).

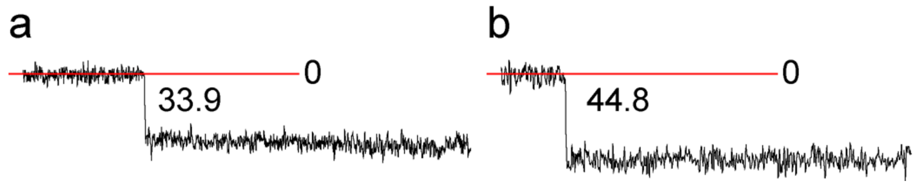

Figure SN2. The value of current is different depending on the orientation of the pore in the bilayer.

Another case in which two pores inserted in bilayer *B* and one pore in bilayer *A*, i.e., configuration (2B,1A), the current calculated using equations (2) and (4) is equal to -44.3 pA which is in agreement with the current step of 44.8 pA (**Figure SN2b**). If the configuration was (1B, 2A) then the expected current would be 45.7 pA.

References

1 Bayley, H. *et al.* Droplet interface bilayers. *Mol Biosyst* **4**, 1191-1208 (2008).
2 Soskine, M., Biesemans, A., Maeyer, M. D. & Maglia, G. Tuning the size and properties of ClyA nanopores assisted by directed evolution. *J Am Chem Soc* **135**, 13456-13463 (2013).
3 Hwang, W. L., Holden, M. A., White, S. & Bayley, H. Electrical behavior of droplet interface bilayer networks: experimental analysis and modeling. *J Am Chem Soc* **129**, 11854-11864 (2007).
